# An organizational perspective on the long-term sustainability of a nursing best practice guidelines program: a case study

**DOI:** 10.1186/s12913-015-1192-6

**Published:** 2015-12-03

**Authors:** Andrea R. Fleiszer, Sonia E. Semenic, Judith A. Ritchie, Marie-Claire Richer, Jean-Louis Denis

**Affiliations:** Ingram School of Nursing, McGill University, Montreal, Canada; McGill University Health Centre (MUHC), Montreal, Canada; École nationale d’administration publique (ENAP), Montreal, Canada

**Keywords:** Sustainability, Program, Organizational change, Innovation, Clinical guidelines, Quality improvement, Management, Leadership, Case study, Nursing

## Abstract

**Background:**

Many healthcare innovations are not sustained over the long term, wasting costly implementation efforts and often desperately-needed initial improvements. Although there have been advances in knowledge about innovation implementation, there has been considerably less attention focused on understanding what happens following the early stages of change. Research is needed to determine how to improve the ‘staying power’ of healthcare innovations. As almost no empirical knowledge exists about innovation sustainability in nursing, the purpose of our study was to understand how a nursing best practice guidelines (BPG) program was sustained over a long-term period in an acute healthcare centre.

**Methods:**

We conducted a qualitative descriptive case study to examine the program’s sustainability at the nursing department level of the organization. The organization was a large, urban, multi-site acute care centre in Canada. The patient safety-oriented BPG program, initiated in 2004, consisted of an organization-wide implementation of three BPGs: falls prevention, pressure ulcer prevention, and pain management. Data were collected eight years following program initiation through 14 key informant interviews, document reviews, and observations. We developed a framework for the sustainability of healthcare innovations to guide data collection and content analysis.

**Results:**

Program sustainability entailed a combination of three essential characteristics: benefits, institutionalization, and development. A constellation of 11 factors most influenced the long-term sustainability of the program. These factors were innovation-, context-, leadership-, and process-related. Three key interactions between factors influencing program sustainability and characteristics of program sustainability accounted for how the program had been sustained. These interactions were between: leadership commitment and benefits; complementarity of leadership actions and both institutionalization and development; and a reflection-and-course-correction strategy and development.

**Conclusions:**

Study findings indicate that the successful initial implementation of an organizational program does not automatically lead to longer-term program sustainability. The persistent, complementary, and aligned actions of committed leaders, in a variety of roles across a health centre department, seem necessary. Organizational leaders should consider a broad conceptualization of sustainability that extends beyond program institutionalization and/or program benefits. The development of an organizational program may be necessary for its long-term survival.

## Background

### Literature review and knowledge gaps

In healthcare, the issue of sustaining organizational and practice changes is of great concern because many beneficial innovations are not maintained over the long term [1, 2]. Innovation decay wastes implementation investments and results in the loss of often desperately-needed initial improvements [3]. Despite advances in knowledge about innovation implementation (e.g., [4, 5]), there has been considerably less attention paid to understanding what happens following the preliminary stages of change [1, 6]. Further research is necessary to determine what can be done to improve the ‘staying power’ of healthcare innovations.

The literature about the sustainability of healthcare innovations varies significantly in definitions and conceptualizations of the phenomenon [1, 6]. Sustainability has been represented as the endurance of innovation-related benefits, the persistence of routinization or institutionalization of the initial innovation, the continued development of the innovation over time, or as different combinations of these characteristics [7]. Some authors have proposed that comprehensive definitions should integrate all three characteristics [3, 8, 9]. In general, sustainability refers to the period following the initial implementation of an innovation, although there is considerable range in the literature regarding timeframes specified (i.e., from 6 months to several decades following initial implementation) [1, 10, 11].

Reviews of the literature about innovation sustainability in health and social service domains have underscored that conceptualizations of sustainability and related factors differ according to the type of innovation and context [1–3, 6, 10]. For example, these reviews indicate there are differences between how a practice change versus a technological change, or an organizational change versus a larger system change, is sustained. These changes are influenced by differences in: types of providers and teams; nature and organization of their work processes; local and professional cultures; governance systems and infrastructures; and social, cultural, economic, and political forces [1–3, 6, 10]. The interactions between the distinctive properties of an innovation, and the unique circumstances within which an innovation becomes embedded, may determine the most appropriate strategies for sustaining change [2, 12]. Yet, Scheirer [2] and Stirman et al. [1], authors of the innovation sustainability reviews, suggested that coordinated knowledge development about sustainability has been hampered by a lack of differentiation between the types of innovations as well as between the contexts within which innovations have been studied. To guide future research, Scheirer [2] proposed a taxonomy of six intervention types–from interventions implemented by individual providers, to programs requiring the coordination among multiple staff, to broad scale system changes–that could be used to guide a clearer distinction between innovations.

There is a specific need for knowledge about the sustainability of healthcare innovations in the field of nursing, particularly in acute care settings. The majority of nurses work in acute care, where they represent the biggest provider group [13, 14]. Expenditures are also greatest in this sector of the health system [15]. Furthermore, significant challenges remain in keeping clinical practices aligned with best evidence, resulting in the delivery of chronically substandard care [14, 16, 17]. Under these circumstances, organizational initiatives, such as organization-wide practice improvement programs, are needed to support evidence-based nursing as well as to ensure standardized, effective care across institutions [18]. However, there is a lack of understanding about how leaders use organizational innovations to develop institutional capacities for: ensuring high quality practice at the frontline, contributing to overall performance, and responding to health system demands [18–22]. There is thus a clear rationale for further developing the knowledge base about how organizational programs can be better sustained in view of achieving these fundamental institutional functions.

In the context of nursing, we found only a few studies [23–31] conducted on the sustainability of innovations. These studies considered a variety of innovations (e.g., individual nurses’ quality improvement training, unit-based process improvement initiatives, nurses’ use of research evidence) in different contexts (e.g., acute care nursing units, community care settings). Yet, none of them looked specifically at the sustainability of organization-wide nursing practice improvement programs. The most relevant to organization-wide program sustainability was a study that identified factors influencing four-year “Magnet Hospital” certification [26]. Sustainability was characterized in terms of innovation continuation (i.e., institutionalization). The key facilitators for sustaining the initiative at the nursing department level of the hospital were related to the executive leadership and management. These included the leaderships’ commitment to the initiative; focus on quality; involvement of staff; and building of knowledge and leadership capital. The main barriers were financial challenges, leader turnover, and departmental philosophy change.

Although not nursing-specific, other studies have been conducted on the sustainability of organization-wide, multidisciplinary care improvement initiatives in acute care centers [32–42]. The majority of these studies quantitatively evaluated the sustainability of innovation-related patient health (i.e., benefits) or provider compliance (i.e., routinization) outcomes across the organization [32–38]. Although they found that outcomes were sustained, to varying degrees, over periods that ranged from two to seven years, these studies did not address what influenced program sustainability or how sustainability was achieved. Four studies [39–42] used qualitative methods to examine the sustainability of hospital practice improvement programs at one to three years following implementation. This small group of studies also characterized sustainability as the continuation of original program activities (i.e., institutionalization) and identified a range of influencing factors. For example, innovation-related factors included: relevance of the program to organizational needs [40]; effectiveness and credibility of the program [40, 42]; and profile of the program within the organization [42]. Context-related factors included dedicated or creative use of resources [39, 40, 42]; organizational capability [42]; and external requirements [42]. Leadership factors included the actions of leaders and/or champions at several institutional levels [39–41], as well as involvement of an extra-organizational facilitator or monitor [40, 42]. Process factors were the use of improvement methodologies to track performance, modify processes, and increase accountability [40–42]; as well as the integration of the program with other organizational initiatives [40, 42].

Further research on organizational program sustainability is justified as the few studies that have identified factors influencing program persistence were not based on existing theory and used narrow conceptualizations of sustainability. Perhaps due to the typically shorter-term timeframes they studied (i.e., less than three years), there was no consideration of how programs may have developed over time. The studies also centered on the perspective of one type of participant (e.g., chief nurses or program coordinators), neglecting the viewpoints of leaders at different levels of organizational management. Moreover, they provided minimal description about the nature of what was sustained and at what level of the organization (department versus unit), with no exploration of the complex relationships between the innovation, the influencing factors, and the circumstances studied. This makes it difficult to determine how programs come to be sustained. These gaps related to sustaining organizational programs are consistent with the general calls for more in-depth, contextually-based, process-oriented studies that consider broader conceptualizations of sustainability and longer-term trajectories of innovations [1, 3, 6, 10, 43]. These research needs, combined with the fact that there is almost no knowledge about innovation sustainability in nursing contexts [44–46], led us to conduct the following study.

### Purpose

The overall study purpose was to understand how a nursing best practice guidelines (BPG) program was sustained over a long-term period in an acute healthcare centre. The specific research questions for this paper were: at the nursing department level of the organization, 1. How was program sustainability characterized?; 2. What were the factors that most influenced program sustainability?; and 3. How was the program sustained over the long-term?

### Conceptual framework

In preparation for this study, we developed a framework for the sustainability of healthcare innovations based on a broad literature review and concept analysis [7]. We defined “innovation sustainability” as a process that emerges from and succeeds innovation implementation, wherein improvements are maintained, new ways of working become routine, surrounding systems are transformed in support, and the innovation may even be developed, over a period of time appropriate to a given situation [7].

The two central components of the framework are “characteristics” (attributes) of sustainability and “factors” (preconditions) that influence sustainability. Over a period of time, the effect of the relationships between the characteristics of and configurations of factors determines the level of sustainability of an innovation.

The three characteristics of innovation sustainability are: benefits, routinization or institutionalization, and development. “Benefits” pertains to consistent achievement of goals and lasting improvement in positive results. “Routinization” or “institutionalization” (the term used henceforth) is the embedded structures and processes of the innovation that are part of the regular or habitual function of individuals, organizations, and systems. “Development” signifies the evolution of the innovation and/or adaptation of the context within which the innovation is embedded.

Four categories of factors proposed to influence sustainability are: innovation, context, leadership, and process. Innovation factors pertain to the nature of the product, practice, policy, or program that is new to the organization or to a group of individuals at the time of adoption. Contextual influences are due to the environment, setting, situation, or conditions within which the innovation is implemented. Leadership is the formal or informal manager(s) or organizer(s) of a group, with certain authorities, attributes, and actions that influence other people. Process factors refer to series of events, strategies, or activities that lead to a particular result.

## Methods

### Study design

To explore the complex phenomenon of program sustainability in its natural, organizational setting, we used a qualitative descriptive case study approach [47–49]. We examined the BPG program from the executive to the frontline levels of an acute health centre. First, we began by investigating program sustainability at the nursing department level of the organization. We then studied program sustainability across two pairs of embedded, contrasting patient care unit subcases (see [50, 51] for reports of unit-level findings). This paper is focused solely on the findings related to program sustainability at the department level. The “nursing department” included the large group of leaders who held managerial or clinical positions at the various levels of the health centre, from highest nursing executive to those working across more than one nursing unit. These nursing department leaders were responsible for the administration and advancement of nursing services within the organization.

### Study setting and BPG program

The organization was a large, tertiary/quaternary urban academic acute health centre in Canada. It had six hospital sites, eight clinical missions/programs, 47 inpatient units, approximately 1000 staffed beds, and more than 3000 nurses. In 2004, the nursing department launched a patient safety-oriented BPG program. Overall program goals were to improve nursing care and patient outcomes; harmonize practices; expand knowledge translation and performance measurement capacities; and develop nursing leadership competence across the organization. The program consisted of the organization-wide implementation of three Registered Nurses’ Association of Ontario^1^ (RNAO) nursing BPGs: falls prevention, pressure ulcer prevention, and pain management.

At the nursing department level, multiple changes were made to establish the BPG program across the health centre. These included designating program co-chairs, creating a steering committee, formalizing BPG-specific task forces, training volunteer practice change advocates, employing a part-time program coordinator, achieving RNAO “Best Practice Spotlight Organization^1^” program status, and securing financial support from numerous sources. The main implementation activities at the department level were related to education (e.g., organization-wide workshops about BPGs and evidence-informed practice change), equipment (e.g., purchase of BPG-recommended patient care items), and evaluation (e.g., establishment of an annual prevalence survey and modification of BPG-related data collection and reporting systems). At the nursing unit level, the main focus of the program was to improve staff nurses’ performance of patient care and documentation practices based on the BPGs. Department-level BPG task force members and change advocates worked with individual nursing units over two to three month-long periods to support the initial implementations of each of the three BPGs. Those externally-facilitated implementation “start-ups” used a combination of educational sessions, nurse BPG champion support, and audit and feedback activities to help nursing units integrate BPG standards into daily practice.

We selected this organization’s nursing BPG program as the focus of our investigation to ascertain how and why the measureable improvements in program-related patient outcomes had been sustained. Administrative data demonstrated that between 2004 and 2012, the organization as a whole achieved maximal reductions in rates of more than 60 % for pressure ulcers, 30 % for falls, and 30 % for patient reports of moderate to severe pain [52]. There was also evidence of program continuation in the form of infrastructure (i.e., committees, resources) and activity (i.e., sustainability-oriented interventions). Further, there were various potential sources of program-related data available including documents, results of previous studies, and many potential informants who had been involved in the program from the beginning.

### Data sources and characteristics

Data collection occurred between August 2011 and June 2012, eight years after program initiation. Fourteen individual, semi-structured, framework-guided interviews were conducted with organizational key informants who were all female Registered Nurses with department-wide leadership positions. We purposefully selected the informants, seeking representation from the variety of roles in the program (e.g., program co-directors, task force members, coordinator, change advocates); emailed potential participants a study information letter; and followed-up with them by telephone or email. All individuals who were invited agreed to participate in the study. Questions during each interview moved from open queries (e.g., What characterizes the sustainability of the program?; What factors have influenced program sustainability?; How has the program been sustained?) to more specific queries (e.g., How has leadership influenced program development?; Which factors have most influenced program sustainability?) if informants did not spontaneously address some of the main elements of the conceptual framework or to pursue additional detail about important emerging topics. We also reviewed program-related documents and completed observational experiences within the nursing department. Additional characteristics of the data sources are summarized in Table 1.

**Table 1:** Characteristics of data sources

Organizational key informants (*N* = 14)	
Current job title	*n* = 1 Director of Nursing
	*n* = 4 Assistant Directors of Nursing
	*n* = 5 Nursing Practice Consultants
	*n* = 3 Clinical Nurse Specialists
	*n* = 1 Nurse Educator
Highest level of education (degree)	12 Masters, 2 Doctoral
Age (yrs)	2 30s, 2 40s, 7 50s, 3 60s
Average time in profession (yrs)	31 (12–46)
Average time in organization (yrs)	19 (8–33)
Average time in current job (yrs)	6.4 (0.5–15)
Average time of interview (mins)	93 (75–120)
Documents (*N* = > 350)	
• Examples: program implementation plans, educational manuals, policy and procedures guides, meeting minutes, website publications, internal and external communications (i.e., letters, emails), presentations, administrative data summaries, reports, funding applications, job descriptions	
Observations and Exchanges (*N* = > 40)	
• Attendance at nursing department conferences, workshop days, and administrative meetings • Participation in informal interactions and meetings with a variety of health centre stakeholders (e.g., executive to frontline staff, patients/families, and volunteers)	

### Data analyses

Data collection and the preliminary phase of analysis occurred concurrently. The main “unit of analysis” was the BPG program at the departmental level of the organization [53]. We used transcripts of the interviews as the primary data source for the analysis. We attended to participants’ perspectives on the degree of importance of, frequency of reference to, and detail of descriptions about the study topics. We also noted numbers and types of informants citing an issue related to program sustainability. The number of interviews conducted was influenced by data redundancy–when no new information was gleaned from additional interviews [54, 55]. Documentary and observational information was used to enhance data completeness. These sources were used to seek substantiation of findings between the data types, fill in gaps or clarify details from interviews, and review information about organizational context [56]. There was overall consistency between the three data types (i.e., interviews, documents, observations).

NVivo 9/10 software was used to organize, code, and query data. Qualitative content analysis guided our data coding and interpretation [48, 57, 58]. We also drew upon three tactics to structure the overall analysis process: data reduction; data display; and conclusion drawing and verification [59]. Multiple rounds of deductive and inductive analysis included: reading raw data and writing descriptive and reflective notes; coding interview transcripts into a categorization scheme developed from the framework as well as emergent findings; composing a detailed case report that incorporated all raw interview data and additional details from documents and observations; synthesizing report content into tables; and writing descriptive and analytical data summaries.

### Strategies for study rigor

We used Guba and Lincoln’s [54] criteria for qualitative research rigor to guide all aspects of the study. The main strategies to ensure credibility were to draw upon multiple types and sources of data, establish trusting investigator-participant relationships, maintain a master coding list, use multiple data coders, debrief about analysis amongst the research team members, and seek substantiation of findings from some participants. Strategies for dependability included adhering to the research study protocol; keeping a project log; documenting choices made at critical project junctures; maintaining organized paper and electronic databases; and composing detailed findings reports. We sought confirmability by remaining close to participants’ verbatim, sampling participants from a variety of program roles, writing reflective memos, and ensuring data redundancy. We aimed for transferability by providing characteristics of the setting and participants, reporting in-depth descriptions of findings, and using a conceptual framework. This study adheres to the RATS guidelines for qualitative research.

### Ethical considerations

The Research Ethics Board (MUHC-11-036-PSY) of McGill University Health Centre, the participating institution, granted approval of the study protocol. Informants provided informed consent in writing prior to participation. Informants’ participation was voluntary and confidential. Complete anonymity for a few participants could not be ensured because of the unique, identifiable nature of their job roles.

## Results

We have organized our findings to answer each of the three research questions. The questions also correspond with central elements of the proposed framework. First, we report on how program sustainability was characterized by describing what elements of the program were sustained. Next, we summarize the factors that most influenced program sustainability over time. Finally, we provide a detailed examination of the salient relationships between characteristics and factors, as these interactions most accounted for how the program was sustained.

### Characteristics of program sustainability

**Fig. 1:**
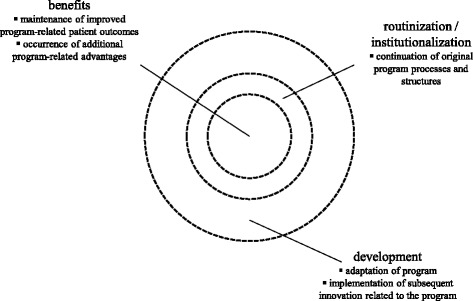
Characteristics of program sustainability at the nursing department level

Informant descriptions reflected that program sustainability consisted of a combination of three essential characteristics: benefits, institutionalization, and development. Informants and documents described substantial program benefit and institutionalization, and provided evidence that, over time, development had become the most prominent feature of program sustainability at the departmental level. We have displayed these three characteristics graphically in Fig. 1.

#### Benefits

The notion of BPG program benefits was described primarily as the improvement in patient outcomes for the organization as a whole, which corresponded to the program’s main objective. Informants cited the administrative data that objectively demonstrated the maintenance of the program’s initial improvements in patient outcomes. However, the majority of informants also contended that the improvements had leveled off over time. As a participant summarized, “the bad news is that we’ve just sustained [results], as opposed to driven the improvements deeper. That’s what we are trying to address now. It’s not good enough to hold gains.” (O13^2^).

Most informants also described the importance of viewing program benefits broadly and across multiple levels of the system. They described other advantages that had occurred beyond improved patient outcomes. These were consistent with other program goals and included increased standardization of the BPG practices across the organization’s nursing units, development of departmental leadership capacity, reinforcement of the organization’s evidence-informed practice culture, and improvement of the department’s performance measurement skills.

#### Institutionalization

Institutionalization was viewed in terms of the program’s enduring processes and structures. Examples of BPG program institutionalization at the organizational level were: program management infrastructure (e.g., co-leaders, steering committee, three BPG task forces, program coordinator); the annual BPG-based, organization-wide prevalence survey (for pressure ulcers and pain) and regular reporting procedure for incidents (falls); a BPG-specific component integrated within the central nursing orientation program; regular topic-specific education sessions for organizational and unit-based nursing staff (e.g., BPG skill workshops); integration of BPG-based documentation items and tools into the health centre’s newly implemented electronic patient record; formalization of BPG-specific organizational policies; and ongoing communication about program activities (e.g., health centre reports and newsletters, international publications). The majority of such changes had been initially instituted in the first few years of the program. Informants perceived these established or recurring program elements as representing the integration of the program into the regular organization and function of the nursing department.

#### Development

Most of the informant and documentary data characterizing program sustainability pertained to development. Development, mainly described in terms of “evolution,” had occurred increasingly in two major ways over the eight years since the start of the initiative. It entailed both the adaptation of the original program as well as the implementation of subsequent related innovation. We provide several illustrations of development in the last section of the findings, as program adaptation and subsequent innovation were central to explaining how the program had been sustained within the nursing department.

### Factors that influenced program sustainability

**Fig. 2:**
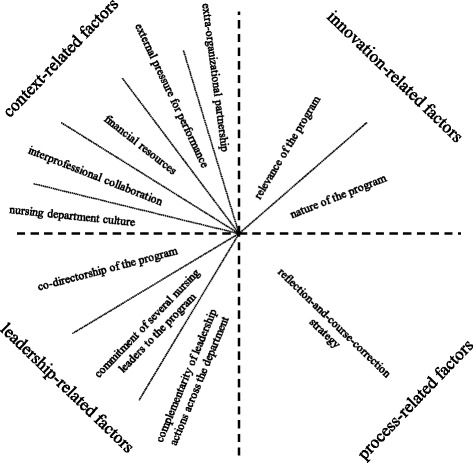
Factors that influenced program sustainability at the nursing department level

Informants described that the influences on program sustainability were “multi-factorial.” Although a variety of factors were discussed, there was consistency in informants’ reports about which innovation, context, leadership, and process factors had been most influential in sustaining the BPG program at the departmental level over time. These 11 factors, sorted into our framework’s four categories of factors, are displayed in Fig. 2. We have also provided informant quotations that illustrate the particular influence of each of these factors. Quotations are presented in Table 2. Informants described that, although negative influences had attenuated program sustainability, positive influences had outweighed the negative to promote program sustainability over the long term.

**Table 2:** Sustainability factors at the nursing department level

Factors	Illustrative quotations	Influence on sustainability
**Innovation-related factors**		
Relevance of the program	• “[The BPG program goals are] a continuing, evolving preoccupation within the organization.” (O09) • “[The program] is an important commitment that focused specifically on the work that nurses do.” (O13) • “The program addressed the issues in a bigger picture way. Not like a band-aid solution, but a longer-term solution.” (O04)	Positive
Nature of the program	• “Now that we have more and more research… we really need to keep it up there. If we’re doing something, there has to be a reason why we’re doing it in a particular way…” (O05) • “It’s our responsibility as an academic nursing group to use evidence.” (O02)	Positive
**Context-related factors**		
Extra-organizational partnership	• “That we were a [RNAO] ‘Spotlight Organization’ (designation) sent a message that this is something big.” (O06)	Positive
External pressure for performance	• “We now have some external benchmark data that we’ve never had before, that shows that we’re not as good as we thought we were. And so we can make more progress. That kind of pressure…” (O11) • “In Canada there’s going to be some more benchmarking, and hospitals comparing themselves… not only the [university health centre] group, but something bigger. So we know that we have to improve.” (O06)	Positive
Financial resources	• “Financially, [the nursing department’s] hands are tied. … We don’t have a budget [for the program], but we still need to do that job. It becomes more and more difficult.” (O05)	Negative
Interprofessional collaboration	• “Although we talk about it being interprofessional, it was a lot of around nursing.” (O12) • “Why aren’t some of the other disciplines–pharmacy, medicine, physiotherapy…–involved anymore? They were participating more at the beginning, or when we needed them…They’re no longer there at the table.” (O09)	Negative
Nursing department culture	• “It’s the culture. The [nursing department] hasn’t really formed its own… I don’t know how successful we’ve been in taking up a sort of identity that we can be proud of.” (O10) • “We do not have that culture of zero tolerance (for substandard care). Think we’re ready to take that responsibility? Far from that.” (O04)	Negative
**Leadership-related factors**		
Co-directorship of the program	• “The working closely together between practice and research [leadership] was also a factor…” (O12) • “[The director of nursing] is trying to put this kind of structure of … best practice guidelines… and making it better for the patient… She’s imposing a structure that we never had.” (O01) • “[The research director] was at arm’s length… could ask good questions… non-territorial… a well-informed voice” with “willingness to path-find.” (O13)	Positive
Commitment of several nursing leaders to the program	• “It’s the ongoing presence of a core team of really committed nursing leaders… who have been involved for a long, long time… People who really took it seriously.... relentless, pushing, never giving up.” (O02) • “The other thing that’s kept it going is the commitment of the leaders. I think if they weren’t so stubborn, and didn’t continue to pull people together, and didn’t continue to push [the program would not have been sustained].” (O11)	Positive
Complementarity of leadership actions across the department	• “Everybody has to see it as important in their work, because it’s all like a chain. And if one [chain link] is weak … then obviously the rest could fall.” (O06) • “If you want to sustain change, you have to have leaders working at various levels of the organization. It’s textbook out of sustainability 101.” (O02)	Positive
**Process-related factor**		
Reflection-and-course-correction strategy	• “The idea of continually revisiting and almost reshaping [the BPG program] is the name of the game” (O10) • “We cannot sit on it and pretend because we’ve changed… it’s a done deal. It never ends…” (O04)	Positive

#### Innovation-related factors

Informants perceived that two main innovation-related factors had positively influenced program sustainability. First, they affirmed that the program had remained relevant to a fundamental mandate of the health centre–to improve the quality of basic patient care. Second, they suggested that the evidence-informed nature of the program (i.e., BPG content and change implementation processes) had continued to be valued by nursing department members.

#### Context-related factors

There were five main context-related factors that influenced program sustainability. Two factors had positive influences. First, informants reported that the formal extra-organizational partnership with RNAO brought prestige to the initiative and encouraged some accountability for program activities and results. Second, in later years of the program, there was increased availability and exchange of patient safety-related data from other local and international centers. This, combined with accreditation bodies’ increased expectations for safety performance, provided external pressure on organizational leaders to ensure program continuation.

The remaining three context-related factors had negative influences. First, informants described that in the absence of a designated operating budget to support the program, it had been a constant challenge to secure sufficient funding for ongoing activities. Second, informants described that issues related to organizational culture had made it difficult to instill a sense of ownership of the program across the nursing department. They indicated that the lack of a unified identity (due in part to the multi-site configuration of the health centre), as well as a weak philosophy of accountability (due to in part to the lack of enforced performance expectations), were entrenched cultural challenges. Third, because collaborations with other professions and departments had not been preserved or expanded over time, the program had remained largely limited to nursing, despite the necessity for multidisciplinary involvement.

#### Leadership-related factors

There were three main leadership-related factors that all had positive influences on program sustainability. The first was that the program had been led by co-directors. Informants emphasized how the same two active and persevering nurse leaders, who held top organizational positions, had chaired the program steering committee from the beginning. Informants reported that the main advantage of the partnership had been that each individual possessed a distinct expertise as well as a unique perspective on the program. One leader had a background in practice and administration; the other was seasoned in research and evidence-informed change. The second positive leadership-related factor was the long-standing commitment of several nursing leaders to the program. The third factor pertained to the complementary nature of those leaders’ actions across the departmental levels.

#### Process-related factors

Informants and documents described a variety of strategies and events that had occurred over the years since the beginning of the program. However, the data clearly revealed one dominant process that had become a strong positive influence on program sustainability. The persisting strategy of “reflection and course-correction” emerged as the main process underlying the use of several sustainability-oriented activities. As the expression suggests, this process entailed iterations over time of leaders’ deliberate efforts to learn from program experiences and, in response, to try to implement continued improvements to the program.

Informants consistently described that the last three factors mentioned above: commitment of leadership, complementarity of leadership actions, and strategy of reflection-and-course-correction, had the greatest positive influences on program sustainability over the long term. We elaborate on these three factors in the next section of the findings, as their influences were central to explaining how the program was sustained.

### How the program was sustained: key relationships between characteristics and factors

**Fig. 3:**
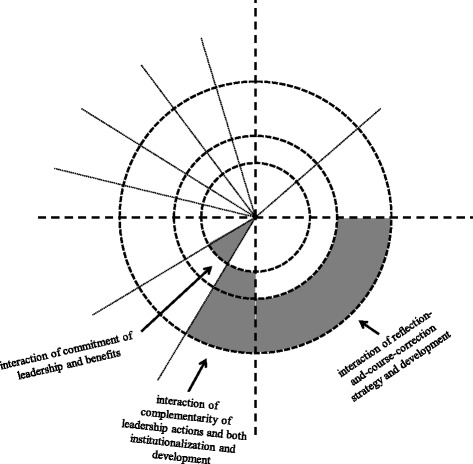
Key interactions between characteristics of and factors that influenced program sustainability at the nursing department level

Although we have summarized separately the characteristics of and the factors that influenced BPG program sustainability, it was descriptions about the complex relationships between characteristics and factors that provided the explanations for how the program had been sustained within the nursing department. Informant accounts of how the program had been sustained converged on three important interactions between characteristics and factors. These three interactions are displayed in Fig. 3, which represents an overlay of Fig. 1 (sustainability characteristics) and Fig. 2 (sustainability factors). Each of these three key interactions is detailed below.

#### Interaction of commitment of leadership with benefits

A key positive leadership factor for program sustainability was long-time commitment of a core group of nursing department leaders to the program. Several leaders, including co-directors, task force members, change advocates, and the BPG program coordinator had “stuck with” the program since early implementation. Informants explained that the leaders had remained dedicated largely because of the multiplicity of benefits that had resulted from the program. Several informants made clear that, as one expressed, “the positive things that we can see have driven the sustainability of us continuing to be involved. If we didn’t feel like we were making progress, the willingness to devote time [to the program] would be zilch” (O11). There was a reinforcing and reciprocal interaction between this sustainability characteristic and factor as leadership commitment contributed to program benefits, and multiple benefits then motivated further leadership commitment.

#### Interaction of complementarity of leadership actions with both institutionalization and development

Another key positive leadership factor for program sustainability was the complementarity of several leaders’ actions across the different departmental levels from the executive to the nursing units. Informants cited the concerted work of a core group of leaders from three particular types of roles–program co-directors, task force members, and clinical program nursing directors. The co-directors led the program from an executive-level perspective. Some task force members, who knew the clinical content of the BPGs, were regularly present on units to observe BPG practices and serve as expert resources for staff. These members then used their unit-based experiences to inform their task force’s strategic departmental work. The impact of some of the clinical program nursing directors was related to their function as the main linkage between the department executive and the nursing unit management teams under their supervision. We have summarized, in Table 3, the informant and documentary descriptions about the role-specific sustainability-oriented leadership actions. These actions, both managerial and clinical in nature, often served multiple purposes at once (e.g., reminding, mandating, educating, supporting).

**Table 3:** Leadership actions across levels of the nursing department

Leadership role	Examples of leadership actions
Program Co-Directors	• Prioritized program activities • Pushed for more BPG-related improvement • Sought creative sources for funding • Advanced an agenda of expectations related to BPGs • Reinforced a BPG-inspired framework for nursing practice • Nurtured a “climate of inquiry” within the department
BPG Task Force Co-Leaders and Other Members	• Facilitated “re-implementation” of BPGs on units with lower levels of sustainability • Implemented more recommendations from each of the BPGs • Developed BPG-based patient teaching materials • Adapted BPG nursing tools to unique patient populations • Engaged other professionals to work on collaboration-related BPG practice challenges • Updated organizational policies and procedures based on BPGs
Clinical Program Nursing Directors	• Included BPGs as a standing agenda item at program meetings • Relieved unit managers from dossiers that took time away from BPG-promoting work • Worked with unit managers to prioritize unit-based BPG practice monitoring • Discussed BPG-related performance data formally and informally with unit management teams • Reminded these teams to use measured BPG-related outcomes as “balancing indicators” during other unit changes • Included BPG-related unit performance as a criterion in unit managers’ individual evaluations or annual unit progress reporting • Participated in the annual health centre-wide prevalence survey

Several informants explained that the significance of the set of actions was that they were interdependently related to one another and orchestrated to operationalize common objectives across the different departmental levels. They were also coherently aligned to address the various sustainability-related issues that arose as the program continued. For example, the program co-directors advanced an agenda of expectations related to BPGs. This led towards task force members updating organizational policies and procedures with BPG content. Accordingly, clinical program directors included BPG-related criteria in their evaluations of managers’ and units’ overall performance. Another example included co-directors prioritizing program activities, resulting in clinical program directors relieving managers from other less essential dossiers. Informants perceived that these coordinated leadership actions promoted aspects of both institutionalization and development. In turn, further institutionalization and development allowed for subsequent iterations of these complements of leadership actions.

#### Interaction of reflection-and-course-correction strategy with development

Interview and documentary data clearly and consistently pointed towards one fundamental sustainability-oriented process that emerged over time. The strategy of “reflection and course-correction” meant that leaders continually assessed the “lessons learned” from their work on the program. Leaders then used these lessons to inform their modifications to the program. This was described as: “you never finish. We got to here, we’ve learned this, now let’s make it better. That is what helps in keeping [the program] alive. It’s evolving” (O12). Although departmental leaders had never overtly planned or named this as a strategy, informants described how several leaders had been deliberate in encouraging a reflective and responsive approach towards the work of sustaining the program.

Informants described several examples of how this process factor (i.e., reflection-and-course-correction strategy) interacted with the development characteristic of sustainability (i.e., program adaptation and subsequent innovation). This interaction was perceived as having been strong and critical to program survival. Below are two commonly-cited illustrations of the relationships between the reflection-and-course-correction strategy and program adaptation. These are followed by the most prominent example related to subsequent innovation. These descriptions depict development at different time periods of the program and show the back-and-forth effects between reflection-and-course-correction and development.

##### Program adaptation

The first example pertained to the adaptation of the initial implementation phase of the program. It took six years for the department to implement the BPGs across almost all of its nursing units. Most informants described how the protracted time period had offered leaders the opportunity to reflect on their experiences of facilitating change with the first cohorts of units and to use the reflections to improve implementation with the later cohorts. One course-correcting change was that the support provided by external facilitators to nursing units became more structured (e.g., they used a more detailed implementation plan). Another course-correcting change was related to the adaptation of the BPGs to the unique circumstances of units (i.e., they varied nature or number of recommendations implemented). Eventually, instead of using standardized implementation formats for all units, “mini implementations” were done on units (e.g., psychiatry) where the BPGs were less relevant. “Tailored implementations” were done on units where a common type of patient sub-population (e.g., those suffering from dementia) prevented direct adherence to the standard BPG recommendations (e.g., ask the patient about pain).

The second example pertained to the adaptation of approaches to monitoring performance and improving quality. The department’s annual prevalence survey (pressure ulcers and pain) and the reporting system for incidents (falls) were the program’s regular performance monitoring activities. Given the leaders’ intents to learn from previous efforts, measurement activities were modified over time to become more comprehensive and sophisticated. Informants and documents noted three course-correcting changes that reflected this. First, leaders made patient outcome performance targets increasingly specific over time. Initially the objective was simply “to improve.” Then leaders set internal benchmarks that felt reasonable for the institution. Leaders eventually drew upon emerging nationally and internationally reported research and administrative publications as standard-setting sources. Second, survey methods were modified over time. Initially, upper-level nursing leaders were the main surveyors. Then increasing numbers of staff nurses were engaged to collect data on units that were not their own. Eventually, in an effort to increase accountability for the BPG practices, staff nurses were required to survey their own units. The information collected in the survey was also changed. It grew from a focus on the three BPGs to also including data about related patient safety practices (e.g., restraints, medications, “sitters”), in parallel with the department’s expanding safety agenda. Third, leaders tried to improve the communication about performance data. Early on, survey results were emailed to clinical program nursing directors and unit managers, under the assumption that the data would be acted on. Eventually, the program co-directors and coordinator scheduled meetings with management teams to discuss results. Informants described that unit teams were going to have to formally report back to their supervisor about how they would respond to their results.

Informants described how the refinement of performance monitoring methods had allowed departmental leaders to identify units with lower levels of sustainability of nursing practices changes and of patient outcomes. This stimulated individualized, departmentally-supported quality improvement initiatives for those particular units. For example, externally-facilitated “BPG re-implementations” were done to help revitalize faded practices. “Targeted support” initiatives, such as assistance with audit and feedback activities, were also provided to help build units’ capacities to more effectively sustain the practice changes.

##### Subsequent innovation

The third example involved subsequent innovation. Several years into the BPG program, leaders’ reflections led them to seek innovative ways to further its initial successes. They chose to implement “Transforming Care at the Bedside^3^” (TCAB), which became the department’s next major nursing practice improvement initiative. TCAB focused on teamwork and critical thinking, patient-centered safety, and value-added nursing care. Nursing unit teams were taught process improvement methodologies like “plan-do-study-act” cycles. Although the spotlight gradually shifted away from the BPG program, unit teams were encouraged to integrate their BPG-related issues into the TCAB initiatives as a means of sustaining the BPGs.

Informants reported that TCAB had become the new focus for several reasons. Leaders had recognized the eventual need for many units to address larger quality issues, further than just the particulars of the BPGs. Leaders also recognized a need for units to work more on unit-specific issues, not just on organizationally-imposed mandates; and for units to build local quality improvement skills, instead of depending on outside support from the department. Most informants explained how TCAB, as one informant articulated, was “centered on broader areas than just nursing practice. It was stepping up the change a few levels…” However, it was a very clear message from informants that they “would not have seen TCAB if BPG hadn’t been here first” (O03). Several described that the development of the department (e.g., increased skill at facilitating organization-wide change, growth of leadership competence, improved performance monitoring capability) that had occurred as a result of the BPG program had “paved the way” for the subsequent, ambitious nursing initiative to be launched. TCAB was viewed by many as the next chapter in the development of the nursing department.

## Discussion

Overall, the findings of this study support our conceptual framework. The framework proposed three characteristics of sustainability (i.e., benefits, institutionalization, and development), influences from four categories of factors (i.e., innovation, context, leadership, and process), and relationships between characteristics and factors. As our findings suggest, some aspects of the framework were more prominent than others in accounting for the sustainability of a BPG program at the nursing department level of the organization. Our discussion is therefore focused on these.

First, with respect to the characterization of program sustainability, it was evident that all three characteristics were present, in different proportions and in different relationships to one another over the eight-year period. This substantiates a conceptualization of innovation sustainability that is broader than just the continuation of initial program activities. The eventual dominance of development thus characterized long-term program sustainability largely in terms of ongoing change. As there has been little examination of the nature and extent of the developmental aspect of sustained healthcare innovations [1], our findings provide some understanding of this characteristic. Development may be regarded as both the adaptation of the initial innovation, as well as the implementation of subsequent related innovation. Our identification of the dominance of development is consistent with some evidence purporting that long-term sustainability is ultimately about continuing change [60–62]. However, a nuance in our findings was that benefits and institutionalization appeared to be precursors to, and remained necessary for development to occur. A sequential relationship between institutionalization, benefits, and development is alluded to in some definitions of innovation sustainability (e.g., [63]), yet the literature has not explicitly considered conceptions of successions or hierarchies between the three characteristics. Furthermore, the presence of at least some of each of the three features, over a long term perspective, is also reflected in our findings. This is consistent with characterizations of sustainability that describe a degree of linkage or interrelationship between the three characteristics as the ultimately desired result of enduring change [3, 64]. For example, we described aspects of institutionalization (i.e., regularity of performance monitoring activities) and of benefits (i.e., recognition of improved patient outcomes). In combination, these aspects prompted the adjustment of program goals, and ultimately contributed toward development (i.e., evolution of the program to achieve even further improvement). Such an example provides evidence for an association between the three characteristics. It also suggests that development may necessary for ensuring “appropriate” sustainability through its relationships with benefits and institutionalization. This contrasts notions of sustainability that are based solely on the institutionalization of program activities, where “inappropriately” engrained changes could in fact occur in the absence of benefits and development.

Second, with respect to factors, we identified 11 factors that had most influenced the sustainability of the BPG program at the nursing department level of the organization. These factors are similar to those previously identified in studies about sustaining organizational programs in acute care [39–42]. While those studies did not identify key factors, we found a constellation of three factors that had the strongest, consistently positive influences on long-term program endurance. These included the: commitment of several nursing leaders, complementarity of leadership actions, and strategy of reflection-and-course-correction. We discuss each of these below.

Leadership, although represented in a range of ways (i.e., informal champion roles, personal attributes, formal leadership positions, management behaviors), is one of the most commonly cited influences across sustainability studies from different fields [1, 3, 11]. Study findings corroborate that commitment and stability of leadership are necessary for sustaining nursing initiatives [26, 27]. Several studies have also identified the influence of one or a few champions as crucial to the sustainability of a variety of innovations [1, 11]. We found, in contrast, that several nursing leaders working collectively, in a complementary fashion across organizational levels were perceived to have been essential for program sustainability within the department. This echoes key findings from two other nursing studies about sustaining culture change initiatives. Those studies emphasized the importance of integrating leadership roles across levels and ensuring coherence between various leadership actions [26–28]. Some authors have proposed that such “plurality,” as in “layers of leadership” or “dispersed leadership,” may be most pertinent for overall organizational performance, especially in large health care settings such as hospitals [3, 65, 66]. We suggest that there seemed to be a cumulative effect of the actions of a coordinated, critical mass of leaders on BPG program sustainability. Our findings also specified the distinct behaviors that organizational nursing leaders used. These nursing leadership actions (presented in Table 3), which had influences both vertically and horizontally across the department, and which were both managerial and clinical in nature, have not been previously described in relation to sustaining organizational programs over the long term. We wish also to draw attention to the BPG program’s co-directorship model which was found to be effective. Stetler et al. [27] similarly noted how duo partnership, shared between two executive leaders, was a factor for the institutionalization of evidence-based practice within hospitals. This further substantiates the potential value of shared leadership models that may benefit from complementarity of expertise, and from the division of the challenging and plentiful work required for leading program sustainability within similar contexts.

Strategic organizational processes, such as the notion of “navigating competing priorities,” have been identified as important for sustaining healthcare innovations [1]. However, such strategies were not described in the few studies pertaining to program sustainability in acute care. This is likely due to the short timeframes considered and their lack of in-depth focus on long-term processes. In their theoretical discussion of innovation sustainability, Chambers et al. [64] proposed that for innovations to be sustained within constantly shifting contexts, learning must be “a core value” underpinning organizational processes. In a similar sense, our findings revealed the compelling effect of a process of learning and modification. This strategic process had guided leaders’ use of sustainability-oriented activities such as those related to initial implementation, performance monitoring, and quality improvement. These are three activities that have been identified to have strong, positive influences on the sustainability of a variety of healthcare innovations [7]. The reflection and course-correction strategy described in our study exhibits features of the phenomenon of organizational learning, wherein organizations undertake strategic processes in an attempt to harmonize continuity and change in view of continual improvement [67–69]. There is some evidence in the literature to support the premise that organizations are more successful at sustaining innovations when learning and adaptation are prominent [3, 64, 70].

Finally, although sustainability characteristics and factors have typically been presented as discrete and distinct (e.g., [1, 11, 71]), our findings illustrate some important and intricate relationships among them. For example, we identified strong interactions between three key factors and each of three characteristics of sustainability. Consequently, based on the findings of this study, the one-way impact of factors on characteristics implied in the initial framework would be more accurately depicted by a two-way arrow indicating reciprocal relationships between characteristics and factors that are made apparent in the Fig. 3 overlay. Such an understanding of sustainability is more consistent with “system-dynamic” conceptualizations that propose complex interdependencies between the multiple elements of sustainability [10, 27, 40, 43, 72]. These complex relationships, which we have illustrated in our study findings, may ultimately help to explain the mechanisms underlying how innovations come to be sustained.

### Methodological strengths and limitations

Although our study provided an in-depth, contextualized, theory-informed illustration of one healthcare organization’s experience of sustaining a program over an eight-year-long term, the investigation would have been strengthened by comparisons with other similar programs within or outside of the organization. It would have been valuable to include the viewpoints of other types of program stakeholders, such as patients, families, or other professionals and administrators, beyond those of a diversity of informants from all levels of the nursing department. Although we used multiple data types, especially history-tracing documentation and long-involved informants, the retrospective approach misses observations attainable only through prospective methods.

### Implications for theory and research

We have provided a theoretical illustration of our findings that is represented by the layered series of Figs. 1, 2 and 3. The graphics concentrate on characteristics and factors, the two main components of the study’s original guiding conceptual framework for the sustainability of innovations. Characteristics (Fig. 1) are represented as three concentric and interrelated circles. Benefits is at the core, indicating its centrality to sustainability, and development is at the periphery, intimating its potential boundlessness. The relative magnitude of each layer may fluctuate depending on the effects of the positive or negative influences of the innovation, context, process, and leadership factors at different phases in time throughout the lifecycle of the innovation. Dotted lines depict the non-discrete relationships between the components. Factors (Fig. 2) are represented as wedges of the circle, divided into innovation-, context-, leadership-, and process-related sections. Each wedge indicates the specific factors that were found to be most influential. In Fig. 3, we highlighted the elements of the framework that were most salient in this study. This kind of theoretical representation of long-term program sustainability, which centers on the relationships between characteristics and factors, could be used to organize results of future studies or plan for sustainability-oriented initiatives. We caution that the static nature of such diagrams does not adequately portray the dynamics of the relationships that we have attempted to animate in the descriptions of findings from this organizational case study. However, the diagrams provided a means for us to represent the multi-layered and interactive complexity of the most significant issues related to sustaining a nursing BPG program in an acute health centre context.

Future research should further explore the characteristics of and the factors related to both the sustainability *and* the discontinuation of different types of innovations in a variety of organizational nursing settings. This could include a focus on organizational changes that are externally mandated, but not perceived to be a local priority; initiatives that are more “bottom-up” instead of organizationally-driven; and programs that have less departmental leadership infrastructure and support. Such investigations could be considered among institutions with varied governance approaches, organizational cultures, and types of healthcare service. We recommend that attention be directed to better understanding the mechanisms underlying the relationship between the three fundamental characteristics of sustainability, as well as between the characteristics and the factors that occur over long term periods–especially those between the development of innovations and organizational learning processes. We recommend further research using qualitative and mixed methodologies including in-depth, longitudinal, comparative case studies and ethnographies. This is appropriate given the complex and evolving nature of the phenomenon of healthcare innovation sustainability, in addition to the early state of knowledge about innovation sustainability in nursing.

### Implications for practice

Sustainability requires considerable attention and continual effort, which should be undertaken as an integrated part of improving overall institutional performance. The persistent, complementary, and shared actions of committed leaders, in a variety of roles across the department, seem necessary. Leaders may need to continually reflect on the successes and failures of their sustainability-oriented work, remaining attentive to the multiplicity of factors that accentuate or attenuate program sustainability, and to use those reflections as a basis for continued evolution and improvement. Structured facilitation and contextually-sensitive program implementation efforts, performance monitoring, and targeted quality improvement efforts with individual units, appear to contribute to program sustainability. Leaders could implement subsequent innovations that build upon, are consistent with, and extend the initial program, in order to respond to changing organizational needs, circumstances, and developmental stage.

## Conclusions

This study is a first to provide an in-depth description of the longevity of an evidence-informed program at the nursing department level of an acute healthcare centre. Our findings suggest that the successful initial implementation of an organizational program does not automatically lead to longer-term program sustainability. Organizational leaders should consider a broad conceptualization of sustainability, beyond just program institutionalization and/or program benefits, because the development of a departmental program may be necessary for its long-term survival.

## Abbreviations

BPG: Best practice guidelines
RNAO: Registered Nurses’ Association of Ontario
TCAB: Transforming Care at the Bedside

## Footnotes

1 RNAO (www.rnao.ca) is the professional association representing Registered Nurses in Ontario, Canada. RNAO develops and publishes evidence-based nursing best practice guidelines (BPGs) that are syntheses of research and other types of evidence. The intention of RNAO’s “Best Practice Spotlight Organization” initiative is to provide some support to organizations that have agreed to implement and evaluate multiple RNAO BPGs.

2 Identifier used for informants: for example, O13 = organizational informant #13.

3 Transforming Care at the Bedside (www.ihi.org/Engage/Initiatives/Completed/TCAB) is an initiative that was developed by the Robert Wood Johnson Foundation and the Institute for Healthcare Improvement. It is centered on the improvement of the quality of care in hospitals. The framework for change is built around improvements in four main categories: safe and reliable care, vitality and teamwork, patient-centered care, and value-added care processes.
